# Lossless Reversible Data Hiding in Encrypted Image for Multiple Data Hiders Based on Pixel Value Order and Secret Sharing

**DOI:** 10.3390/s23104865

**Published:** 2023-05-18

**Authors:** Haoyang Yu, Junwei Zhang, Zixiao Xiang, Biao Liu, Huamin Feng

**Affiliations:** 1 School of Cyberspace Security, Beijing University of Posts and Telecommunications, Beijing 100876, China; 2 Beijing Electronic Science and Technology Institute, Beijing 100070, China; 3 School of Cyber Engineering, Xidian University, Xi’an 710071, China

**Keywords:** secret sharing, secure multi-party computing, reversible data hiding in encrypted domain

## Abstract

Reversible data hiding in encrypted images (RDH-EI) is instrumental in image privacy protection and data embedding. However, conventional RDH-EI models, involving image providers, data hiders, and receivers, limit the number of data hiders to one, which restricts its applicability in scenarios requiring multiple data embedders. Therefore, the need for an RDH-EI accommodating multiple data hiders, especially for copyright protection, has become crucial. Addressing this, we introduce the application of Pixel Value Order (PVO) technology to encrypted reversible data hiding, combined with the secret image sharing (SIS) scheme. This creates a novel scheme, PVO, Chaotic System, Secret Sharing-based Reversible Data Hiding in Encrypted Image (PCSRDH-EI), which satisfies the (k,n) threshold property. An image is partitioned into *N* shadow images, and reconstruction is feasible when at least *k* shadow images are available. This method enables separate data extraction and image decryption. Our scheme combines stream encryption, based on chaotic systems, with secret sharing, underpinned by the Chinese remainder theorem (CRT), ensuring secure secret sharing. Empirical tests show that PCSRDH-EI can reach a maximum embedding rate of 5.706 bpp, outperforming the state-of-the-art and demonstrating superior encryption effects.

## 1. Introduction

Reversible data hiding (RDH) has evolved into a compelling methodology, facilitating the embedding of confidential data within various forms of media. This spans sectors such as military, medical, national governance, and copyright-protected content [1]. Traditional RDH research, conducted in the realm of plaintext, primarily revolves around three data embedding techniques: histogram shifting [2], difference expansion [3], and lossless compression [4]. The overarching objective of these methodologies is to augment the embedding rate while enhancing the visual quality of the carrier images.

In summary, Reversible Data Hiding (RDH) technology is a robust tool for reversibly embedding confidential data without damaging the original carrier. Its applications are expansive, and it proves particularly beneficial in sensitive sectors where data security is imperative. However, for content-sensitive scenarios, it is necessary to employ encryption techniques to develop an Encrypted Domain Reversible Data Hiding (RDH-EI) method, suitable for information-sensitive situations. These encryption techniques secure image content by transforming the original image into an unintelligible version using an encryption key.

RDH in encrypted images (RDH-EI) is especially advantageous in sensitive fields where data security is paramount. Despite its limitation in multiple data hider scenarios, the inherent reversibility of RDH technology makes it an optimal choice for situations demanding zero tolerance for image loss. An encrypted domain RDH-EI method, suitable for content-sensitive scenarios, can be developed using encryption techniques.

The traditional RDH-EI model restricts the number of data providers and data hiders to a single entity, limiting its applicability in multiple data hider scenarios. However, the crucial feature of reversibility permits perfect reconstruction of the original carrier during covert data extraction. This attribute renders RDH technology an ideal choice for scenarios necessitating zero tolerance for image loss, such as satellite imagery in the military, government images in judicial determinations, and medical imaging in healthcare.

RDH-EI is a subset of Reversible Data Hiding in Encrypted Domain (RDH-ED) [5], effectively resolves the issue of embedding and extracting confidential data from encrypted images. In this technique, the encrypted image data serves as the carrier. The data can be embedded into the encrypted image without causing any pixel loss in the carrier image during extraction.

**Figure 1:**
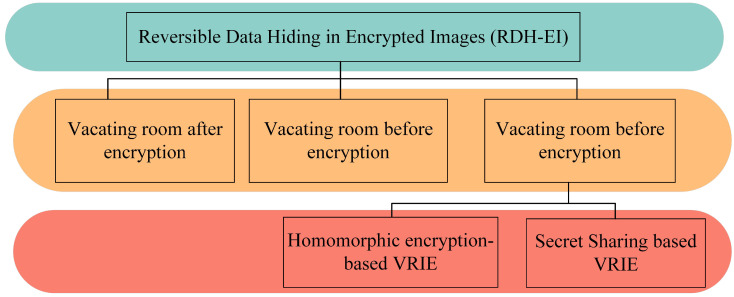
Literature review of RDH-EI, in which three categories of reversible data hiding with encryption methods can be identified: Vacating Room After Encryption (VRAE), Vacating Room Before Encryption (VRBE), and Vacating Room In Encryption (VRIE).

As shown in Figure 1, according to the differences in secret data embedding models, we divide RDH-EI into three categories.

(a) Vacating Room After Encryption (VRAE). In the framework of VRAE, Puech et al. [6] first proposed the method of vacating room after AES encryption, in which additional data can only be extracted according to the local standard deviation of the image before decryption of the token image; subsequently, Zhang et al. [5] vacated space by flipping the three lowest significant bits after encryption based on stream ciphers, and secret data can only be extracted using the fluctuation function defined by the local characteristics of the image after decryption of the token image; in order to achieve the separability of the algorithm, Zhang et al. [7] first proposed a separable scheme by losslessly compressing the ciphertext image to generate redundancy, but its embedding capacity is low.
(b) Vacating Room Before Encryption (VRBE). The VRBE mode is used to generate redundancy by using image correlation or other pre-processing operations before image encryption, and its implementation methods are mainly based on lossless compression [8], pixel prediction [9,10,11], and frequency domain transformation [12].
(c) Vacating Room In Encryption (VRIE). (1) Homomorphic encryption-based VRIE. Zhang et al. [13] proposed the RDH-EI scheme based on VRIE by quantizing the encrypted domain after LWE encryption and using the redundancy generated by ciphertext expansion to embed secret data; Huang et al. [14] used prediction error to free up redundant space during stream cipher encryption to enhance the embedding capacity. In recent years, Chen et al. [15] proposed an algorithm based on Paillier public key encryption, which uses the homomorphic property to embed information in the ciphertext domain, and the decrypted plaintext still maintains the relevant properties of the embedded information, but there is partial pixel overflow after embedding the information; Wu et al. [16] improved the method of the literature [15] by solving the overflow problem, and the security of the homomorphic encryption algorithm relies on long keys, which increases the computational overhead and brings about severe data scaling. (2) Secret Sharing-based VRIE. Secret sharing techniques are widely applied in edge computing [17], data sharing [18], and outsourcing computing [19]. Thien and Lin [20] first proposed the concept of secret image sharing in 2002, which is based on the idea of secret sharing (SS) proposed by Shamir [21] in 1979. This technique can effectively solve the data extension problem of the RDH-EI algorithm based on homomorphic encryption, and Wu et al. [22] first proposed the RDH-ED algorithm based on secret sharing encryption, which splits the carrier image into multiple shadow images of the same size as the carrier image with secret sharing encryption, and then embeds the secret data into the shadow images by means of difference expansion and prediction error histogram translation. The problems of high encryption overhead and serious data expansion are effectively solved. As an important multi-party secure cryptosystem, this method uses a threshold function to address important data shares multiplied by different shares stored at different users. Chen et al. [23] further reduced the time complexity by embedding secret data into a pair of pixels using the additive homomorphism property of multiple secret sharing and combining the difference expansion. Ke et al. [24] proposed a separable RDH-ED based on the Chinese residue theorem separable RDH-ED, which achieves separability by combining two embedding methods. In 2022, Xiong et al. [25] proposed an RDH-EI scheme, which uses Asmuth-Bloom’s secret sharing scheme based on the CRT to divide the pixels into several secret share subgraphs, but the embedding efficiency (0.5 bpp) of this method still has room for optimization.

To solve the problems of low embedding efficiency, low embedding capacity, complex auxiliary information for embedding, and the limited number of embedding parties in RDH-EI, we propose a lossless RDH-EI method for multiple data hiders based on PVO and secret sharing. Compared with the existing RDH-EI based on secret sharing, we put forward a new method for the first time, which combines an Arnold cat map and a logistic equation [26] with a CRT-based secret sharing scheme and adds PVO technology, which improves the embedding rate and encryption efficiency while improving security. In addition, unlike other RDH-EI that requires auxiliary information and guiding images, the proposed method in this paper does not need to send guiding images. However, the data hider can automatically convert the shadow image into the guide image by using the data embedding key, which can significantly improve the embedding efficiency.

Main contributions of this paper. This paper describes the main contributions of a lossless RDH-EI (Reversible Data Hiding in Encrypted Images) method for multiple data hiders, based on PVO (Pixel Value Order) and secret sharing. The following are the key contributions of the proposed method:

Novel PCSRDH-EI Method: This paper introduces an innovative lossless RDH-EI method, which allows for multiple data hiders to embed data in encrypted images without any loss. This method is based on the PVO chaotic system and secret sharing, achieving a maximum embedding rate and surpassing existing methods, as well as significantly improving encryption efficiency and effect.

Enhanced Security with Combined Techniques: Combining stream encryption and secret sharing technology, this paper ensures a secure environment for data hiders to embed their data without fear of leaks. Additionally, the proposed scheme guarantees lossless data embedding and extraction, preserving the original image digital assets and enabling full recovery.

No Extra Guide Parameters Required: The proposed scheme does not require any extra guide parameters. By embedding/extracting the key, the data hider can transform the shadow image into an guide image that does not expose the original content information, thereby improving the usability of the scheme.

Organization of this paper. We divide our paper into five sections. Section 1 describes the research area and the existing schemes’ shortcomings and details the paper’s main contributions. Section 2 presents the specific details of the proposed scheme. In Section 3, we give a precise analysis and demonstration of our scheme. Section 4 presents the scheme’s effectiveness and a detailed comparison with the state-of-the-art encryption techniques. In Section 5, we give a summary and outlook of our work.

## 2. PCSRDH-EI Method

**Figure 2:**
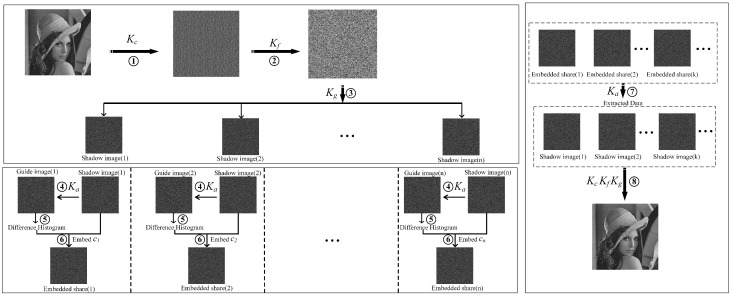
The framework of our scheme. The scenario of our method has one image owner, at least three data hiders, and at least one receiver. The main steps are ① coarse-grained encryption, ② fine-grained encryption, ③ shadow image generation, ④ guide image generation, ⑤ difference generation, ⑥ data embedding, ⑦ embedded data extraction, and ⑧ carrier image recovery.

In this section, we provide a detailed description of our proposed method. As depicted in Figure 2, our model involves three key participants: an image owner, a minimum of three data hiders, and at least one receiver. The primary stages of our method encompass image encryption, shadow image creation, data embedding, data extraction, and carrier image recovery.

During the Image Encryption phase, the image owner divides the image into blocks and implements coarse-grained disruption encryption between these blocks. They further apply fine-grained encryption algorithms and the PVO algorithm within the blocks to enhance the security of the encrypted image.

In the Shadow Image Generation phase, the image owner partitions the encrypted image into numerous shadow images and distributes them to a predetermined number of data hiders. This measure ensures that no individual data hider can access the entire image, thereby augmenting the security of the scheme.

During the Data Embedding stage, each data hider employs the embedding secret key to produce the embedding guide map and embeds the confidential data within their respective shadow images. This procedure ensures the dispersion of the embedded data across multiple shadow images, which further bolsters the scheme’s security.

In the Embedded Data Extraction and Image Recovery phase, the receiver employs the secret extraction key to retrieve the embedded data from the shadow images. Upon amassing a minimum of ’k’ secret shares, the receiver can flawlessly recover the carrier image by integrating these shares with the encryption key.

### 2.1. Image Encryption

The image owner splits a carrier image of size H×W into non-overlapping sub-blocks of size 2×2 and the number ⌈H/2×W/2⌉, where H is the height of the image and W is the width of the image. $i_{h,w}$ is the pixel value at location (h,w), where $I_{h,w}\in \left[0,255\right],1\leq h\leq H,1\leq w\leq W$. Each block has a number in order, numbered in the range 1,2,...,⌈H/2×W/2⌉. This section divides encryption into two main steps: coarse-grained and fine-grained. The encryption unit of coarse-grained encryption is a 2 × 2 non-overlapping sub-block, the encryption algorithm is Arnold’s cat map, and the target of fine-grained encryption is the four pixels inside the non-overlapping sub-block, which is implemented as Algorithm 1.

**Algorithm 1** Image encryption.Input: The original image I(H×W), coarse-grained encryption key $K_{c}\leftarrow \left(p,q\right)$, fine-grained encryption key $K_{f}\leftarrow \left(\eta ,x_{0},\forall \right)$.Output: The encrypted image $I^{\prime }$  1. Initialize h←0,w←0,s←0,t←0  2. Use $K_{c}$ to encrypt the original image I by Equation (2) and obtain $I^{*}$  3. Use $K_{f}$ to generate the fine-grained encryption stream σ by Equation (3)  4. while h≤H do  5.       While w≤W do  6.             $I_{h,w}^{\prime }=I_{h,w}^{*}+\left(\sigma_{s,t}\;\mathrm{mod}\;256\right)+256$  7.             w←w+1  8.             s=⌊h/2⌋, t=⌊w/2⌋  9.       end while10. h←h+111. s=⌊h/2⌋, t=⌊w/2⌋12. end while13. Return $I^{\prime }$

Secret key definition. The implementation of encryption or decryption methods, as described in the work by [27], requires both the sender and the recipient of the image to possess a secret key SK. This unique key consists of two parts: a coarse-grained encryption key denoted as $K_{c}\leftarrow \left(p,q\right)$ and a fine-grained encryption key denoted as $K_{f}\leftarrow \left(\eta ,x_{0},\forall \right)$. This secret key is generated from 65 hexadecimal digits (260 bits), ranging from P1 to P52, and used to compute the initial conditions and control parameters of the chaotic mapping using the expression indicated below:

(1) $$\begin{array}{cc} K_{c1} & ={\left(P_{1},P_{2},\ldots ,P_{13}\right)}_{10}\\ K_{c2} & ={\left(P_{14},P_{15},\ldots ,P_{26}\right)}_{10}\\ K_{f1} & =\frac{{\left(P_{27},P_{38},\ldots ,P_{39}\right)}_{10}}{2^{52}+1}\times 10^{-8}\\ K_{f2} & =\frac{{\left(P_{40},P_{41},\ldots ,P_{52}\right)}_{10}}{2^{52}+1}\\ K_{f3} & ={\left(P_{53},P_{54},\ldots ,P_{65}\right)}_{10} \end{array}$$

In which $K_{c1}$, $K_{c2}$, $K_{f3}$ belong to $\left(0,2^{52}\right)$, and $K_{f1}$, $K_{f2}$ belong to (0,1).

Coarse-grained encryption. Before the image owner splits the image into shadow images, the image needs to be scrambled to ensure its security. Since the fine-grained encryption only needs to satisfy the randomness to generate the scrambling table, and the test sets used in this paper are square matrix, i.e., H=W, this paper uses a two-dimensional Arnold’s cat map to generate the scrambling table. For the case of H≠W, other similar random number sorting algorithms can be used to generate the scrambling table, which this paper will not discuss. The image owner uses the coarse-grained encryption key $K_{c}\leftarrow \left(p,q\right)$ as the secret seed key and performs

(2) $$\left[\begin{array}{c} x_{n+1}\\ y_{n+1} \end{array}\right]\leftarrow \left[\begin{array}{cc} 1 & p\\ q & pq+1 \end{array}\right]\left[\begin{array}{c} x_{n}\\ y_{n} \end{array}\right]\;\mathrm{mod}\;N$$

where $\left(x_{n},y_{n}\right)$ is the position coordinates of the image block in the original image, $\left(x_{n+1},y_{n+1}\right)$ is the transformed position, mod is the modulus operation, and *N* is the size of the image; the image must be square. Otherwise, it does not have the condition of the Arnold transform, for rectangular images can also be converted into a square way for topological reasoning. Each coordinate has a unique new coordinate corresponding to it after permutation, which means that each image block has a unique position to mark the position after permutation, and the image encryption procedure is formulated by

1≤t≤⌈H/2×W/2⌉, which is the t-th encrypted image block. *I* is the carrier image and $I^{*}$ is the coarse-grained encrypted image.

Fine-grained encryption. In the proposed scheme, there is no restriction on the specific scrambling method, and we take the enlarged 1D logical mapping as an example. 1D logical mapping only needs to save the seed key, the enlargement factor, and the length of the generated 1D stream, which significantly saves the storage space and the efficiency of crucial transmission. The image owner uses the fine-grained encryption key $K_{f}\leftarrow \left(\eta ,x_{0},\forall \right)$ to generate a random stream by

(3) $$x_{r+1}=\eta x_{r}\left(1-x_{r}\right)$$

Furthermore, the generated sequence $x=\left(x_{1},x_{2},\cdots x_{\left\lceil H/2\times W/2\right\rceil }\right)$ is enlarged by a factor of ∀ and rounded down, to obtain the size ⌈H/2×W/2⌉, and perform the following encryption operation:

(4) $$I_{h,w}^{\prime }=I_{h,w}^{*}+\left(\sigma_{s,t}\;\mathrm{mod}\;256\right)+256$$

where $I_{h,w}^{\prime }$ is the result of the image owner’s encryption using the key $K_{f}$, where *i* and *j* denote rows and columns, respectively, where h=1,2,…,H,w=1,2,…,W. $I_{h,w}$ and $I_{h,w}^{\prime }$ are the ciphertext and plaintext at (h,w), respectively.

Moreover, $T_{f_{s,t}}$ is the key corresponding to the (s,t) th image block in $T_{f}$, where s=[h/2],t=⌈w/2⌉, at the block level, the image owner uses fine-grained encryption by

(5) $$I^{\prime }\left(I^{\prime (1)},I^{\prime (2)},\cdots ,I^{\prime (\lceil H/2\times W/2\rceil )}\right)=Enc_{\mathrm{fine}\;}\left(I^{*},K_{f}\right)$$

where $I^{*}$ is the block-level data after coarse-grained encryption, $I^{\prime }$ is the block-level data after coarse-grained encryption, and 1≤t≤⌈H/2×W/2⌉ is the t-th encrypted image block.

### 2.2. Shadow Image Generation

Since the encryption algorithm in this paper employs a permutation algorithm between blocks, there is no scrambling algorithm for pixels between blocks. The RDH algorithm based on histogram shifting (HS) can meet the user’s needs. When the data hider receives the encrypted share, the secure multi-party embedding is performed under the guidance of the dealer. The data distributor can collect encrypted secret data and initiate the embedding phase in a practical application with multiple users and messages. By involving data distributors, communication between multiple data hiders can be avoided. Data hiding based on histogram embedding can be performed on encrypted domains, achieving the high visual quality of the tagged images. Watermark embedding is divided into three main steps. Algorithm 2 demonstrates the detailed algorithmic process in the form of pseudo-code.

**Algorithm 2** Shadow image generation.Input: The encrypted image $I^{\prime }$, share generation key $K_{g}=\left(\vartheta ,Mb\right)$, number of shadow images *n*Output: The shadow images τ, position correspondence table ϖ  1. Initialize h←0,w←0  2. while h≤H/2 do  3.       While w≤W/2 do  4.             $\delta_{1}\leftarrow x\left[h,w\right]$, $\delta_{2}\leftarrow x\left[h+1,w\right]$, $\delta_{3}\leftarrow x\left[h,w+1\right]$, $\delta_{4}\leftarrow x\left[h+1,w+1\right]$  5.             Record the original position $\left[\delta_{1},\delta_{2},\delta_{3},\delta_{4}\right]$ in ϖ[h,w]  6.             Sort x[h,w] by Equation (6) to get $\left\{\delta_{1}^{\prime },\delta_{2}^{\prime },\delta_{3}^{\prime },\delta_{4}^{\prime }\right\}$ and $x^{\prime }\left[h,w\right]$  7.             Computes $d_{\max}=\delta_{4}^{\prime }-\delta_{3}^{\prime }$, $d_{\min}=\delta_{1}^{\prime }-\delta_{2}^{\prime }$  8.             w←w+1, t=⌈w/2⌉  9.      end while10. h←h+1, s=⌈h/2⌉11. end while12, while h≤H do13.       While w≤W do14.             While i≤n do15.                   $\;\tau_{i}\left(h,w\right)=\vartheta \left(x\left(h,w\right)\right)\;\mathrm{mod}\;Mb^{\left(i\right)}$16.                   i←i+117.             end while18.             w←w+119.      end while20.      h←h+121. end while22. Return τ

#### 2.2.1. Pixel Value Order

Intra-block ascending mapping. For a block of 2×2 pixels $\delta =(\delta_{1},\delta_{2},\delta_{3},\delta_{4})$, the image owner computes the new block of pixels utilizing an ascending ordering algorithm:

(6) $$\left(\delta_{1}^{\prime },\delta_{2}^{\prime },\delta_{3}^{\prime },\delta_{4}^{\prime }\right)\leftarrow \mathrm{PVO}\left(\delta_{1},\delta_{2},\delta_{3},\delta_{4}\right)$$

where the set $\left(\delta_{1}^{\prime },\delta_{2}^{\prime },\delta_{3}^{\prime },\delta_{4}^{\prime }\right)$ to the set $\left(\delta_{1},\delta_{2},\delta_{3},\delta_{4}\right)$ is a full projection and satisfies

(7) $$\delta_{1}^{\prime }\leq \delta_{2}^{\prime }\leq \delta_{3}^{\prime }\leq \delta_{4}^{\prime }$$

Therefore, the two largest values $\delta_{4}^{\prime }$ and $\delta_{3}^{\prime }$ can predict the value of the other with the value of one, and $\delta_{1}$ and $\delta_{2}^{\prime }$ can also predict the value of the other with the value of one, and their prediction differences are

(8) $$\left\{\begin{array}{c} d_{\max}=\delta_{4}^{\prime }-\delta_{3}^{\prime }\\ \;d_{\min}=\delta_{1}^{\prime }-\delta_{2}^{\prime } \end{array}\right.$$

$d_{\max\;}$ and $d_{min}$ are the maximum and minimum prediction errors in the image chunks, respectively. For the result of $I^{\prime }$ after the fine-grained encryption of the image, and after repeating the same PosCon-operation, we can obtain $d_{\max\;}$ and $d_{min}$ for all chunks of the whole image, and calculate the histograms of the maximum prediction error and minimum prediction error accordingly.

In addition, the image owner uses 00,01,10,11 to denote the original pixel positions (1,2,3,4) matched by the scrambled pixels, respectively, and saves the original position correspondence table ϖ corresponding to the scrambled image pixels.

#### 2.2.2. Key Generation

Shadow recovery key generation. For the secret sharing scheme with a $\left(k,n\right)$ threshold, we choose a set of modules $Mb=\left\{{Mb}^{\left(1\right)},\;{\;Mb}^{\left(2\right)},\cdots ,{Mb}^{\left(n\right)}\right\}$, where the elements in Mb satisfy $\left\{128<{Mb}^{\left(1\right)}<{Mb}^{\left(2\right)}<\cdots <{Mb}^{\left(n\right)}\leq 251<Mp\right\}$, satisfying the following conditions:

(9) $$\gcd\left(Mb^{\left(i\right)},Mb^{\left(j\right)}\right)=1,i\neq j$$

(10) $$\gcd\left(Mb^{\left(j\right)},Mp\right)=1,i=1,2,\ldots ,n$$

(11) $$Mp=\prod_{i=1}^{k}Mb^{\left(i\right)}$$

In addition, we set the secret key ϑ to satisfy the following relation:

(12) $$\vartheta \in Z_{\left(\prod_{i=1}^{k}Mb^{\left(i\right)}\right)\left(2^{8}-1\right)}$$

(13) $$\gcd\left(Mb^{\left(i\right)},\vartheta \right)=1,i=1,2,\ldots ,n$$

Then, the shadow image is generated by $K_{g}\leftarrow \left(\vartheta ,Mb\right)$.

Embedding key generation. The randomly chosen integer ϑ is the secret key, and using the equivalence property of congruence since ϑ is the amplification parameter, the following two calculations have equivalence conditions under modb:

(14) xϑmodb⇔x(ϑmodb)modb

Therefore, firstly, ϑ is converted into the residue form of $a_{i}=\vartheta \;mod\;{Mb}_{i}$, and the secret parameter ϑ can be guaranteed not to be revealed without exposing a certain number of ${Mb}_{i}$. The generated key is $K_{a}\leftarrow \left(\mathrm{Mb},a,\Delta ,\chi \right),\Delta$ is the threshold parameter which determines the embedding payload, pixels exceeding the threshold Δ are panned, and those within the threshold Δ are embedded. The size of Δ will affect the visual quality of the labeled image. For example, when increasing, the embedding payload will increase, but the visual quality will decrease.

#### 2.2.3. Shadow Image Generation

Unlike the scheme proposed by Xiong et al. [25] which only adds large random numbers (only addition), the secret sharing scheme used in this paper uses the method of adding perturbations first and then scaling up (a combination of addition and multiplication) to improve security. For a single pixel value, x(h,w),h=1,2,…,H,w=1,2,…,W, an image of size H×W, and the encryption key $K_{g}=\left(\vartheta ,Mb\right)$, let us calculate

(15) $$\begin{array}{c} \vartheta \left(x\left(h,w\right)\right)=\tau_{1}\left(h,w\right)\;\mathrm{mod}\;Mb^{\left(1\right)}\\ \vartheta \left(x\left(h,w\right)\right)=\tau_{2}\left(h,w\right)\;\mathrm{mod}\;Mb^{\left(2\right)}\\ \ldots \\ \vartheta \left(x\left(h,w\right)\right)=\tau_{n}\left(h,w\right)\;\mathrm{mod}\;Mb^{\left(n\right)} \end{array}$$

where $\tau_{1}\left(h,w\right)\;\;\tau_{2}\left(h,w\right),\ldots ,\tau_{n}\left(h,w\right)$ represent the secret shares split by the pixel values at position (*h*,*w*) in the image, and the *n* secret shadow images after splitting $\left(\tau^{\left(1\right)},\tau^{\left(2\right)},\cdots ,\tau^{\left(n\right)}\right)$ are sent to the corresponding data embedders ${DH}^{\left(i\right)}$ for i=1,2,…,n.

### 2.3. Data Embedding

Since the same noise is added to each image sub-block during fine-grained encryption, the differences between pixels do not change, which means that additional information can be embedded in the DH embedding data using homomorphic embedding. A module homomorphic embedding approach is adopted in this paper, in which any shadow image after image segmentation is used as a carrier. The additive homomorphic property of secret sharing is taken advantage of so that the data hider can embed all extra data into the shadow image independently. This algorithm can extract the extra data rapidly from the newly generated shadow image. It is convenient for multiple users to tag, manage, and retrieve secret texts independently too improve the embedding efficiency, i.e., to embed as much data as possible with fewer modifications. Importantly, each data hider owns an independent shadow image and can produce the embedding instruction and data embedding images independently. Consequently, the embedding capacity scales linearly with the number of data hiders, such that the overall capacity is obtained by multiplying the capacity of a single image by the number of hiders involved. Algorithm 3 demonstrates the detailed algorithmic process in the form of pseudo-code.

**Algorithm 3** Distributed data embedding.Input: The secret share of the i-th embedding party $\tau_{i}$, data embedding key $K_{a}\leftarrow \left(\mathrm{Mb},a,\Delta ,\chi \right)$, data to be embedded $c_{i}\leftarrow \left\{c_{i_{0}},c_{i_{1}}\right\}$.Output: The i-th embedded shadow image $S_{i}$  1. Initialize h←0,w←0  2, while h≤H/2 do  3.       While w≤W/2 do  4.             $\delta_{1}\leftarrow \tau_{i}^{\prime }\left[2h,2w\right]$, $\delta_{2}\leftarrow \tau_{i}^{\prime }\left[2h+1,2w\right]$  5.             $\delta_{3}\leftarrow \tau_{i}^{\prime }\left[2h,2w+1\right]$, $\delta_{4}\leftarrow \tau_{i}^{\prime }\left[2h+1,2w+1\right]$  6.             ${\dot{\delta }}_{j}=\delta_{j}a^{\left(i\right)}\;\mathrm{mod}\;Mb^{\left(i\right)},j=1,2,3,4$  7.             Compute $d_{\max}\left[h,w\right]$, $d_{\min}\left[h,w\right]$ by Equations (17) and (18)  8.             Embed $c_{i_{1}}\left[h,w\right]$ into $\delta_{4}^{\prime }$ by Equation (19) and $c_{i_{0}}\left[h,w\right]$ into $\delta_{1}^{\prime }$ by Equation (20)  9.             $S_{i}\left[2h,2w\right]\leftarrow \delta_{1}^{\prime }$, $S_{i}\left[2h+1,2w\right]\leftarrow \delta_{2}$10.             $S_{i}\left[2h,2w+1\right]\leftarrow \delta_{3}$, $S_{i}\left[2h+1,2w+1\right]\leftarrow \delta_{4}^{\prime }$11.             w←w+112.      end while13. h←h+114. end while15. Return $S_{i}$

Upon receiving the image to be embedded $\tau^{\left(i\right)}$, ${DH}^{\left(i\right)}$ divides it into non-overlapping 2×2 sub-blocks. We denote the block at position (h,w) as $\left\{\delta_{1}\left[h,w\right],\delta_{2}\left[h,w\right],\delta_{3}\left[h,w\right],\delta_{4}\left[h,w\right]\right\}$ (for ease of writing, we abbreviate it as $\left\{\delta_{1},\delta_{2},\delta_{3},\delta_{4}\right\}$) and embed $c_{i}\left[h,w\right]\leftarrow \left\{c_{i_{0}},c_{i_{1}}\right\}$). Specifically, $c_{i_{0}}\left[h,w\right]$ is embedded into $\delta_{1}$ while $c_{i_{1}}\left[h,w\right]$ is embedded into $\delta_{4}$. To obtain a difference histogram that encodes the image difference information, we employ the secret extraction key $K_{a}$ as follows:

(16) $${\dot{\delta }}_{j}=\delta_{j}a^{\left(i\right)}\;\mathrm{mod}\;Mb^{\left(i\right)},j=1,2,3,4$$

Prediction error recovery. For a single 2×2 pixel block, the four elements within the block perform sum and mode operations using the same cryptographic secret key. This means that the difference between any two pixels within the block should satisfy the ascending ordering, and thus the true prediction error is recovered in the following manner:

(17) $$d_{\max}\left[h,w\right]=\left\{\begin{array}{c} {\dot{\delta }}_{4}-{\dot{\delta }}_{3},if:{\dot{\delta }}_{4}-{\dot{\delta }}_{3}\geq 0\\ {\dot{\delta }}_{4}-{\dot{\delta }}_{3}+Mb^{\left(i\right)},if:{\dot{\delta }}_{4}-{\dot{\delta }}_{3}<0 \end{array}\right.$$

(18) $$d_{\min}\left[h,w\right]=\left\{\begin{array}{c} {\dot{\delta }}_{1}-{\dot{\delta }}_{2},if:{\dot{\delta }}_{1}-{\dot{\delta }}_{2}\leq 0\\ {\dot{\delta }}_{1}-{\dot{\delta }}_{2}-Mb^{\left(i\right)},if:{\dot{\delta }}_{1}-{\dot{\delta }}_{2}>0 \end{array}\right.$$

The difference histograms are mainly concentrated around the value of 0, and the prediction error statistics of ciphertext images are the same as those of plaintext images, so the prediction error statistics of ciphertext images retain the statistical characteristics of plaintext. The prediction error histogram of a raw image is generally Laplacian, so we can use the histogram shifting technique to hide the data in the ciphertext image.

Histogram embedding. We perform the translation embedding in two prediction error histograms of the ciphertext image. It is worth noting that, in addition to the 0 value, several histograms of its nearby values are also high, so we utilize multiple histogram embeddings to increase the embedding capacity. In addition, due to the unique nature of module homomorphic secret sharing in this paper, the selected module bases are less than 252. Therefore, the data overflow before secret sharing will not affect the embedding flags and results after secret sharing in the all-position embeddable histogram translation embedding method.The image owner can use an additional auxiliary information table to cooperate with data embedding and extraction. Therefore, the histogram panning embedding method based on secret sharing can effectively solve the problem of possible overflow and underflow during the data panning process.

We set the storage threshold at Δ, pixels exceeding the threshold Δ are shifted, those within the threshold Δ are embedded, and according to the prediction error $d_{\max\;}$, the data $c_{1}$ are embedded by shifting the maximum value $\delta_{4}$, the maximum value of possible embedding is χ, and the data embedding algorithm embed(τ) is executed, so the data embedding is performed by

(19) $$\delta_{4}^{\prime }\leftarrow \left\{\begin{array}{cc} \delta_{4}+c_{1}\left[h,w\right]\Delta & \;\mathrm{if}\;0\leq d_{\max\;}\left[h,w\right]\leq \Delta \\ \delta_{4}+\chi \Delta & \;\mathrm{else}\; \end{array}\right.$$

According to the equation prediction error $d_{\min\;}$, embedding the data $c_{0}$ by shifting the minimum $\delta_{1}$, the data embedding is performed by

(20) $$\delta_{1}^{\prime }\leftarrow \left\{\begin{array}{cc} \delta_{1}-c_{0}\left[h,w\right]\Delta & \;\mathrm{if}\;-\Delta \leq d_{\min}^{\left(i\right)}\leq 0\\ \delta_{1}-\chi \Delta & \;\mathrm{else}\; \end{array}\right.$$

The embedding algorithm is executed on all image blocks of the image to obtain the labeled image $S^{\left(i\right)}=\mathrm{embed}\left(\tau^{\left(i\right)}\right)$, and send it to the corresponding ${\mathrm{receiver}}^{\left(i\right)}\;,i=1,2,\cdots n$.

### 2.4. Data Extraction

When the secret data are embedded into the encrypted shadow share, the receiver can collect the tagged encrypted shadow images from different data hiders. Upon receiving the extraction secret key $K_{a}^{\left(i\right)}$ that corresponds to the ${Share}^{\left(i\right)}$ of the i-th shadow image, the i-th receiver can recover the embedded data individually. At position (h,w) of $S^{\left(i\right)}$, the block is denoted as $\left\{\delta_{1}\left[h,w\right],\delta_{2}\left[h,w\right],\delta_{3}\left[h,w\right],\delta_{4}\left[h,w\right]\right\}$ (for ease of writing, we abbreviate it as $\left\{\delta_{1},\delta_{2},\delta_{3},\delta_{4}\right\}$). We extract $w\left[h,w\right]\leftarrow \{c_{0}^{\prime }\left[h,w\right],c_{1}^{\prime }\left[h,w\right]\}$, wherein $c_{0}^{\prime }\left[h,w\right]$ is extracted into $\delta_{1}$, and $c_{1}^{\prime }\left[h,w\right]$ is extracted into $\delta_{4}$. Moreover, we can obtain $d_{max}\left[h,w\right]$ and $d_{min}\left[h,w\right]$ using the same method as in Equations (16)–(18).

Iterating through all the storage blocks of the block, we obtain the computational prediction error $d_{max}\left[h,w\right]$ and $d_{min}\left[h,w\right]$. From the nature of the 2×2 pixel-based block, it is known that there are two positions within the block where the data can be embedded, and the possible embedded data are ${\tilde{w}}_{0}$ by Equation (21) and $\;{\tilde{w}}_{1}$ by Equation (22).

(21) $$\;{\tilde{w}}_{0}\left[h,w\right]=\left\{\begin{array}{cc} \left\lfloor d_{\max}^{\left(i\right)}\left(h,w\right)/\Delta \right\rfloor & \;\mathrm{if}\;\Delta <d_{\max}^{\left(i\right)}\left(h,w\right)\leq \chi \Delta \;\mathrm{and}\;d_{\min}^{\left(i\right)}\left(h,w\right)/\Delta \neq 0\\ \left(d_{\max}^{\left(i\right)}\left(h,w\right)/\Delta \right)-1 & \;\mathrm{if}\;\Delta <d_{\max}^{\left(i\right)}\left(h,w\right)\leq \chi \Delta \;\mathrm{and}\;d_{\min}^{\left(i\right)}\left(h,w\right)/\Delta =0\\ 0 & \;\mathrm{if}\;0<d_{\max}^{\left(i\right)}\left(h,w\right)\leq \Delta \\ NULL & \;\mathrm{if}\;\chi \Delta <d_{\max}^{\left(i\right)}\left(h,w\right) \end{array}\right.$$

(22) $${\tilde{w}}_{1}\left[h,w\right]=\left\{\begin{array}{cc} \left\lfloor |d_{\min}^{\left(i\right)}\left(h,w\right)/\Delta |\right\rfloor & \;\mathrm{if}\;\Delta <\left|d_{\min}^{\left(i\right)}\left(h,w\right)\right|\leq \chi \Delta \;\mathrm{and}\;d_{\min}^{\left(i\right)}\left(h,w\right)/\Delta \neq 0\\ \left|d_{\min}^{\left(i\right)}\left(h,w\right)/\Delta \right|-1 & \;\mathrm{if}\;\Delta <\left|d_{\min}^{\left(i\right)}\left(h,w\right)\right|\leq \chi \Delta \;\mathrm{and}\;d_{\min}^{\left(i\right)}\left(h,w\right)/\Delta =0\\ 0 & \;\mathrm{if}\;0<\left|d_{\min}^{\left(i\right)}\left(h,w\right)\right|\leq \Delta \\ NULL & \;\mathrm{if}\;\chi \Delta <\left|d_{\min}^{\left(i\right)}\left(h,w\right)\right| \end{array}\right.$$

Iterate over all blocks and recover all embedded data. Algorithm 4 demonstrates the detailed algorithmic process in the form of pseudo-code.

**Algorithm 4** Data extraction.Input: The i-th embedded shadow image $S_{i}$, data extraction key $K_{a}\leftarrow \left(\mathrm{Mb},a,\Delta ,\chi \right)$.Output: The extracted data $c^{\prime }$  1. Initialize h←0,w←0  2, while h≤H/2 do  3.       While w≤W/2 do  4.             $\delta_{1}\leftarrow S_{i}^{\prime }\left[2h,2w\right]$, $\delta_{2}\leftarrow S_{i}^{\prime }\left[2h+1,2w\right]$  5.             $\delta_{3}\leftarrow S_{i}^{\prime }\left[2h,2w+1\right]$, $\delta_{4}\leftarrow S_{i}^{\prime }\left[2h+1,2w+1\right]$  6.             ${\dot{\delta }}_{j}=\delta_{j}a^{\left(i\right)}\;\mathrm{mod}\;Mb^{\left(i\right)},j=1,2,3,4$  7.             Compute $d_{\max}\left[h,w\right]$, $d_{\min}\left[h,w\right]$ by Equations (17) and (18)  8.             Extract $c_{1}^{\prime }\left[h,w\right]$ from $\delta_{4}^{\prime }$ by Equation (21)  9.             Extract $c_{0}^{\prime }\left[h,w\right]$ from $\delta_{1}^{\prime }$ by Equation (22)10.             w←w+111.      end while12. h←h+113. end while14. Return $c^{\prime }$

### 2.5. Image Recovery

Algorithm 5 demonstrates the detailed algorithmic process of image recovery in the form of pseudo-code. After receiving the secret shares of k, the image owner first uses the inverse theorem based on the residual theorem of the Chinese Remainder to obtain the fine-grained encrypted digital image data. For the share of the *h*th row and *w*th column of the image $S_{1}\left(h,w\right),S_{2}\left(h,w\right),\ldots ,S_{k}\left(h,w\right)$, $S_{4}^{\left(i\right)}\left(h,w\right)$ and ${\dot{S}}_{1}^{\left(i\right)}\left(h,w\right)$ can be recovered by

(23) $${\dot{S}}_{4}^{\left(i\right)}\left(h,w\right)=\left\{\begin{array}{cc} S_{4}^{\left(i\right)}\left(h,w\right)-\left\lfloor d_{\max}^{\left(i\right)}\left(h,w\right)/\Delta \right\rfloor \Delta \;\mathrm{mod}\;Mb_{i} & \mathrm{if}\;{\tilde{w}}_{0}=\left\lfloor d_{\max}^{\left(i\right)}\left(h,w\right)/\Delta \right\rfloor \\ S_{4}^{\left(i\right)}\left(h,w\right)-d_{\max}^{\left(i\right)}\left(h,w\right)+\Delta \;\mathrm{mod}\;Mb_{i} & \mathrm{if}\;{\tilde{w}}_{0}=\left(d_{\max}^{\left(i\right)}\left(h,w\right)/\Delta \right)-1\\ S_{4}^{\left(i\right)}\left(h,w\right)\;\mathrm{mod}\;Mb_{i} & \mathrm{if}\;{\tilde{w}}_{0}=0\\ S_{4}^{\left(i\right)}\left(h,w\right)\;-\Delta \chi \mathrm{mod}\;Mb_{i} & \mathrm{if}\;{\tilde{w}}_{0}=NULL \end{array}\right.$$

(24) $${\dot{S}}_{1}^{\left(i\right)}\left(h,w\right)=\left\{\begin{array}{cc} S_{1}^{\left(i\right)}\left(h,w\right)+\left\lfloor |d_{\min}^{\left(i\right)}\left(h,w\right)/\Delta |\right\rfloor \Delta \;\mathrm{mod}\;Mb_{i} & \mathrm{if}\;{\tilde{w}}_{1}=\left\lfloor |d_{\min}^{\left(i\right)}\left(h,w\right)/\Delta |\right\rfloor \\ S_{1}^{\left(i\right)}\left(h,w\right)-d_{\min}^{\left(i\right)}\left(h,w\right)-\Delta & \mathrm{if}\;{\tilde{w}}_{1}=\left|d_{\min}^{\left(i\right)}\left(h,w\right)/\Delta \right|-1\\ S_{1}^{\left(i\right)}\left(h,w\right)\;\mathrm{mod}\;Mb_{i} & \mathrm{if}\;{\tilde{w}}_{1}=0\\ S_{1}^{\left(i\right)}\left(h,w\right)\;+\Delta \chi \mathrm{mod}\;Mb_{i} & \mathrm{if}\;{\tilde{w}}_{1}=\mathrm{NULL} \end{array}\right.$$

The secret can be recovered by executing the following secret recovery algorithm:

(25) $$\begin{array}{c} \tilde{I}\left(h,w\right)=\sum_{i=1}^{k}M_{i}S_{i}\left(h,w\right)M_{i}^{-1}\;\mathrm{mod}\;Mp\\ M_{i}=Mp/Mb_{i}\\ M_{i}M_{i}^{-1}=1\;\mathrm{mod}\;Mp \end{array}$$

**Algorithm 5** Image recovery.Input: The i-th embedded shadow image $S_{i}$, data extraction key $K_{a}\leftarrow \left(\mathrm{Mb},a,\Delta ,\chi \right)$, decryption key $K_{c}$ and $K_{f}$, position correspondence table ϖ.Output: Recovered image  1. Initialize h←0,w←0  2, while h≤H/2 do  3.       While w≤W/2 do  4.             $\delta_{1}\leftarrow S_{i}^{\prime }\left[2h,2w\right]$, $\delta_{2}\leftarrow S_{i}^{\prime }\left[2h+1,2w\right]$  5.             $\delta_{3}\leftarrow S_{i}^{\prime }\left[2h,2w+1\right]$, $\delta_{4}\leftarrow S_{i}^{\prime }\left[2h+1,2w+1\right]$  6.             ${\dot{\delta }}_{j}=\delta_{j}a^{\left(i\right)}\;\mathrm{mod}\;Mb^{\left(i\right)},j=1,2,3,4$  7.             Compute $d_{\max}\left[h,w\right]$, $d_{\min}\left[h,w\right]$ by Equations (17) and (18)  8.             Recover ${\dot{S}}_{4}^{\left(i\right)}\left[h,w\right]$ by Equation (23) and ${\dot{S}}_{1}^{\left(i\right)}\left[h,w\right]$ by Equation (24)  9.             Recover $\dot{S}\left[h,w\right]$ by Equation (25)10.             w←w+111.      end while12. h←h+113. end while14. Recovery *I* by Equation (26)–(28)15. Return *I*

After recovering the secret sub share, a fine-grained decryption algorithm is executed, and it is decrypted by

(26) $$I^{*}\left(h,w\right)=\tilde{I}\left(h,w\right)-256-\left(T_{f_{s,t}}\;\mathrm{mod}\;256\right)$$

where $\tilde{I}\left(h,w\right)$ is the decryption result obtained by the image owner using the secret recovery algorithm, $I_{i,j}$ and $I_{i,j}^{\prime }$ are the (h,w) ciphertext and plaintext, respectively. $T_{f_{s,t}}$ is the key corresponding to the (s,t) image block in $T_{f}$, where s=⌈h/2⌉,t=⌈w/2⌉. After finishing the coarse-grained decryption, the IO executes the coarse-grained decryption algorithm using the key $K_{c}$ and finishes the decryption to obtain the lossless carrier image I:

(27) $$\left[\begin{array}{c} x_{n+1}\\ y_{n+1} \end{array}\right]=\left[\begin{array}{cc} pq+1 & -p\\ -q & 1 \end{array}\right]\left[\begin{array}{c} x_{n}\\ y_{n} \end{array}\right]\;\mathrm{mod}\;N$$

(28) $$I\left(I^{\left(1\right)},I^{\left(2\right)},\cdots ,I^{\left([H/2\times W/2]\right)}\right)={\mathrm{Dec}}_{\mathrm{coarse}\;}\left(I^{*},K_{c}\right)$$

The method proposed in this paper has significant social impact in fields such as military imagery, remote healthcare, and forensic investigation. These fields demand the assurance of data security and integrity, which the proposed method can improve and ensure. As a multi-party secure cryptographic system, it can enhance data disaster recovery through secret sharing properties and have a positive effect on data security.

## 3. Demonstration and Analysis

### 3.1. Method Demonstration

In the last section, we introduced the specific details of our proposed protocol. To better understand the proposed method, in this chapter, we show the whole method in distributions. That is, the image encryption example diagram, secret split example diagram, data embedding example diagram, and data extraction example diagram; additionally, the extraction of feature images is integrated into the overall framework.

We will give a concrete example of the protocol and give an analysis and security proof of the protocol. We will take four 2 × 2 image blocks as an example and set the parameters of (k, n) secret sharing as 4 and 5.

**Figure 3:**
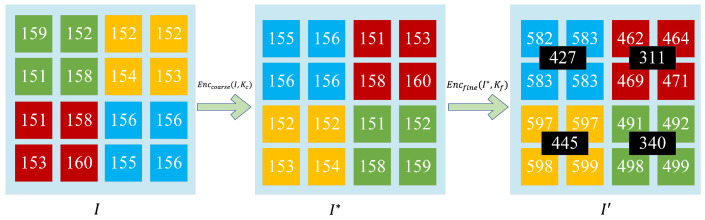
Example of image encryption. The size of the demo image is 4×4, the coarse-grained encryption secret key is (11,10,01,00), and the fine-grained encryption key is (427,311,455,340).

**Image encryption**. As shown in Figure 3, let an image with 16 original pixels be I. The image is divided into four 2 × 2 dot blocks ($I^{\left(1\right)},I^{\left(2\right)},I^{\left(3\right)},I^{\left(4\right)}$), and pixels of the same color are grouped together in the image encryption example diagram:

(29) $$\left\{\begin{array}{c} I^{\left(1\right)}=\left(I_{1}^{\left(1\right)},I_{2}^{\left(1\right)},I_{3}^{\left(1\right)},I_{4}^{\left(1\right)}\right)=\left(159,152,151,158\right)\\ I^{\left(2\right)}=\left(I_{1}^{\left(2\right)},I_{2}^{\left(2\right)},I_{3}^{\left(3\right)},I_{4}^{\left(4\right)}\right)=\left(152,152,154,153\right)\\ I^{\left(3\right)}=\left(I_{1}^{\left(3\right)},I_{2}^{\left(3\right)},I_{3}^{\left(3\right)},I_{4}^{\left(3\right)}\right)=\left(151,158,153,160\right)\\ I^{\left(4\right)}=\left(I_{1}^{\left(4\right)},I_{2}^{\left(4\right)},I_{3}^{\left(4\right)},I_{4}^{\left(4\right)}\right)=\left(156,156,155,156\right) \end{array}\right.$$

The image owner uses the coarse-grained Arnold image permutation algorithm $Enc_{coarse}\left(I,K_{c}\right)$ to obtain $I^{*}$:

(30) $$\left\{\begin{array}{c} I^{*(1)}=\left(I_{1}^{*}\left(1\right),I_{2}^{*(1)},I_{3}^{*(1)},I_{4}^{*}\left(1\right)=\left(155,156,156,156\right)\right.\\ I^{*(2)}=\left(I_{1}^{*(2)},I_{2}^{*(2)},I_{3}^{*(3)},I_{4}^{*}\right)=\left(151,153,158,160\right)\\ I^{*(3)}=\left(I_{1}^{*(3)},I_{2}^{*(3)},I_{3}^{*(3)},I_{4}^{*(3)}\right)=\left(152,152,153,154\right)\\ I^{*(4)}=\left(I_{1}^{*(4)},I_{2}^{*(4)},I_{3}^{*(4)},I_{4}^{*}\left(4\right)=\left(151,152,158,159\right)\right. \end{array}\right.$$

The image owner uses the fine-grained image scrambling algorithm $Enc_{fine}\left(I^{*},K_{f}\right)$, where the encryption key $K_{f}=427,311,455,340$, and obtains $I^{\prime }$:

(31) $$\left\{\begin{array}{c} I^{\prime (1)}=\left(I_{1}^{\prime (1)},I_{2}^{\prime (1)},I_{3}^{\prime (1)},I_{4}^{\prime (1)}\right)=\left(582,583,583,583\right)\\ I^{\prime (2)}=\left(I_{1}^{\prime (2)},I_{2}^{\prime (2)},I_{3}^{\prime (2)},I_{4}^{\prime (2)}\right)=\left(462,464,459,471\right)\\ I^{\prime (3)}=\left(I_{1}^{\prime (3)},I_{2}^{\prime (3)},I_{3}^{\prime (3)},I_{4}^{\prime (3)}\right)=\left(597,597,598,599\right)\\ I^{\prime (4)}=\left(I_{1}^{\prime (4)},I_{2}^{\prime (4)},I_{3}^{\prime (4)},I_{4}^{\prime (4)}\right)=\left(491,492,498,499\right) \end{array}\right.$$

**Figure 4:**
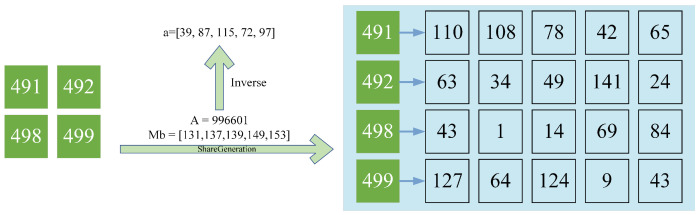
Example of shadow images generation. The random number is 996,601, the module base is (131, 137, 139, 149, 153), and the corresponding inverse is (39, 87, 115, 72, 97).

Shadow image generation: As shown in Figure 4, the image owner randomly picks the module Mb and the random mapping parameter A=996,601, as well as the secret key $K_{a}\leftarrow \left(\mathrm{Mb},a,\Delta \chi \right)$ for the module Mb inverse of the random parameter A:

(32) $$Mb=\left(Mb_{1},Mb_{2},Mb_{3},Mb_{4},Mb_{5}\right)=\left(131,137,139,149,153\right)$$

(33) $$a=A^{-1}\;\mathrm{mod}\;Mb=\left(a^{\left(1\right)},a^{\left(2\right)},a^{\left(3\right)},a^{\left(4\right)},a^{\left(5\right)}\right)=\left(39,87,115,72,97\right)$$

The image owner sends the split secret data $I^{\prime }$ and the embedding secret key $K_{a}$ to the corresponding data hider using a trusted channel. ${\tilde{I}}^{\left(1∼5\right)}$ is the secret share map of the difference information obtained by $DH^{\left(1∼5\right)}$ with $K_{a}^{\left(1∼5\right)}$:

(34) $$\left\{\begin{array}{c} {\tilde{I}}^{\left(1\right)}=\left(I_{1}^{\left(1\right)},I_{1}^{\prime (2)},I_{1}^{\prime (3)},I_{1}^{\prime (4)}\right)a^{\left(1\right)}\;\mathrm{mod}\;Mb^{\left(1\right)}=\left(98,99,105,106\right)\\ {\tilde{I}}^{\left(2\right)}=\left(I^{\prime (1)},I_{2}^{\prime (2)},I_{2}^{\prime (3)},I_{2}^{\prime (4)}\right)a^{\left(2\right)}\;\mathrm{mod}\;Mb^{\left(2\right)}=\left(80,81,87,88\right)\\ {\tilde{I}}^{\left(3\right)}=\left(I^{\prime (1)}{}_{3}^{\left(1\right)},I_{3}^{\prime (2)},I_{3}^{\prime (3)},I^{\prime }{}_{3}^{\left(4\right)}\right)a^{\left(3\right)}\;\mathrm{mod}\;Mb^{\left(3\right)}=\left(74,75,81,82\right)\\ {\tilde{I}}^{\left(4\right)}=\left(I^{\prime }{}_{4}^{\left(1\right)},I_{4}^{\prime (2)},I_{4}^{\prime (3)},I_{4}^{\prime (4)}\right)a^{\left(4\right)}\;\mathrm{mod}\;Mb^{\left(4\right)}=\left(44,45,51,52\right)\\ {\tilde{I}}^{\left(5\right)}=\left(I^{\prime (1)},I_{1}^{\prime (2)},I_{1}^{\prime (3)},I_{1}^{\prime (4)}\right)a^{\left(5\right)}\;\mathrm{mod}\;Mb^{\left(5\right)}=\left(32,33,39,40\right) \end{array}\right.$$

**Figure 5:**
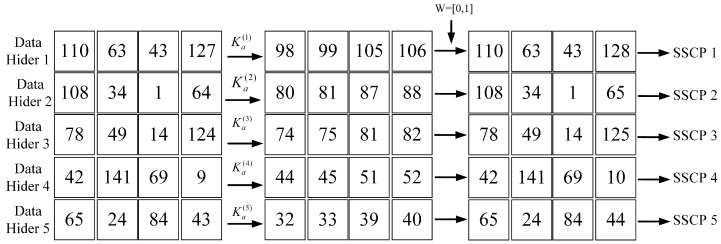
Example of data hiding phase. The secret data to be embedded are (0,1), and the data hider first converts the shadow image into a guide image. Subsequently, it performs an embedding operation on the shadow image based on the difference between the pixels of the guide image.

As shown in Figure 5, we can see that $\left|d_{\max}^{\left(1∼5\right)}\right|=\left|d_{\min}^{\left(1∼5\right)}\right|=1$; both are less than or equal to Δ. Therefore, the data hider performs the data embedding operation for both the highest and lowest bits to obtain the shadow image share after embedding the data and sends the secret share to the corresponding SSCP:

(35) $$\left\{\begin{array}{c} \;\mathrm{Share}{\;}^{\left(1\right)}=\left(110-0\times \Delta ,63,43,127+1\times \Delta \right)=\left(110,63,43,128\right)\\ \;\mathrm{Share}{\;}^{\left(2\right)}=\left(108-0\times \Delta ,34,1,64+1\times \Delta \right)=\left(108,34,1,65\right)\\ \;\mathrm{Share}{\;}^{\left(3\right)}=\left(78-0\times \Delta ,49,14,124+1\times \Delta \right)=\left(78,49,14,125\right)\\ \;\mathrm{Share}{\;}^{\left(4\right)}=\left(42-0\times \Delta ,141,69,9+1\times \Delta \right)=\left(42,141,69,10\right)\\ \;\mathrm{Share}{\;}^{\left(5\right)}=\left(65-0\times \Delta ,24,84,43+1\times \Delta \right)=\left(65,24,84,44\right) \end{array}\right.$$

**Figure 6:**
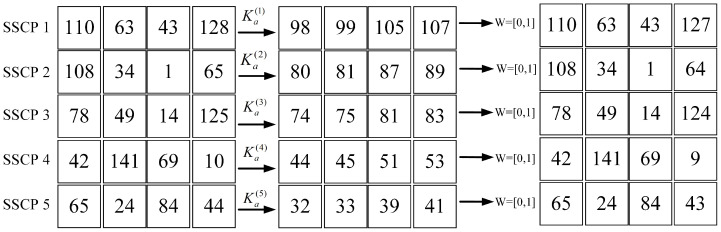
Example of data extraction phase. Receiver first converts the embedded shadow image into a guide image. Subsequently, it performs an data extraction operation on the shadow image based on the difference between the pixels of the guide image, and obtains the secret data (0,1).

As shown in Figure 6, receiver ${}^{\left(1∼5\right)}$ recovers the secret share map that represents the image difference information and obtains W=[0,1] after receiving the embedding data recovery request using $K_{a}^{\left(1∼5\right)}$. The shadow image recovery and image decryption are the reverse of the above steps and will not be repeated.

### 3.2. Method Analysis

**Definition** **1.** *The coarse-fine-grain encryption method combined with PVO performs the encryption and decryption of the image correctly.*

**Proof.**  For an image *I*, let us suppose the initial position of pixel points is $\left(x_{n},y_{n}\right)$; according to the coarse-grained encryption and decryption method is Equations (2) and (27), there is

(36) $$\left[\begin{array}{cc} pq+1 & -p\\ -q & 1 \end{array}\right]\left[\begin{array}{c} x_{n+1}\\ y_{n+1} \end{array}\right]\;\mathrm{mod}\;N=\left[\begin{array}{cc} pq+1 & -p\\ -q & 1 \end{array}\right]\left[\begin{array}{cc} 1 & p\\ q & pq+1 \end{array}\right]\left[\begin{array}{c} x_{n}\\ y_{n} \end{array}\right]\;\mathrm{mod}\;N$$

and there is

(37) $$\left[\begin{array}{cc} pq+1 & -p\\ -q & 1 \end{array}\right]\left[\begin{array}{cc} 1 & p\\ q & pq+1 \end{array}\right]=\left[\begin{array}{cc} 1 & 0\\ 0 & 1 \end{array}\right]$$

It can be easily seen that

(38) $$\left[\begin{array}{cc} 1 & 0\\ 0 & 1 \end{array}\right]\left[\begin{array}{c} x_{n}\\ y_{n} \end{array}\right]=\left[\begin{array}{c} x_{n}\\ y_{n} \end{array}\right]$$

Thus, the coarse-grained encryption is lossless and reversible.

Moreover, the fine-grained encryption uses a one-dimensional logical chaotic system to generate a fixed sequence with the same initial value $x_{o}$ and parameter μ. Therefore, the random numbers added to the image pixel blocks can be recovered by generating the same sequence, so fine-grained encryption is lossless and reversible.

Furthermore, $\left(\delta_{1},\delta_{2},\delta_{3},\delta_{4}\right)\leftarrow PVO\left(\delta_{1},\delta_{2},\delta_{3},\delta_{4}\right)$, where the set $\left(\delta_{1},\delta_{2},\delta_{3},\delta_{4}\right)$ and where the set $\left(\;delta_{1},\delta_{2},\delta_{3},\delta_{4}\right)$ to the set $\left(\delta_{1},\delta_{2},\delta_{3},\delta_{4}\right)$ is a full projection. The position of the original pixel can be restored losslessly according to the position relationship table if the position relationship table is saved with 8 bits. The position of the original pixel can be restored losslessly according to the position relationship table. Therefore, the correctness of the encryption and decryption combined with the coarse–fine-grained encryption method of PVO is proved. □

**Definition** **2.** *This secret-sharing method can correctly split the encrypted image into multiple shadow images and recover the shadow image into the carrier image. The message hider embeds the message, and the receiver retrieves the embedded secret without affecting the lossless recovery of the carrier image.*

**Proof.**  Let the encryption key of the secret sharing algorithm be $K_{g}=\left(\vartheta ,\mathrm{Mb}\right)$, the original data are *x*, and the image owner performs the secret splitting algorithm with Equation (15), where $b_{1},b_{2},b_{3},\cdots b_{k}$ are two mutually prime positive integers, and in the embedding phase of the data hider, there are

(39) $$\left\{\begin{array}{c} \tilde{r_{1}}=r_{1}+\Delta w\left(\;\mathrm{mod}\;Mb_{1}\right)\\ \;\tilde{r_{2}}=r_{2}+\Delta w\left(\;\mathrm{mod}\;Mb_{1}\right)\\ \;\;\vdots \\ \tilde{r_{k}}=r_{k}+\Delta w\left(\;\mathrm{mod}\;Mb_{k}\right) \end{array}\right.$$

There must be a unique solution for the embedded data, i.e.,

(40) $$T=x\vartheta +w\equiv B_{1}B_{1}^{-1}\left(r_{1}+w\right)+\cdots +B_{k}B_{k}^{-1}\left(r_{k}+w\right)\left(\;\mathrm{mod}\;B\right)$$

In case the image owner needs to recover the carrier image, there is

$$x=\frac{B_{1}B_{1}^{-1}\left(r_{1}+\Delta w\right)+\cdots +B_{k}B_{k}^{-1}\left(r_{k}+\Delta w\right)\left(\;\mathrm{mod}\;B\right)-\Delta w}{\vartheta }$$

Thus, the splitting and recovery of secret sharing are non-destructive and reversible. Thus, definition 2 is proved. □

**Definition** **3.** *The proposed scheme in this paper exhibits a threshold property of (k, n), which implies that s colluding parties with less than k information embedders would still fail to reconstruct the images encrypted using PVO’s coarse–fine-grained encryption method.*

**Proof.**  We take the example of each data hider having a single shadow image, assuming there are n data hiders. Let $K_{g}=\left(\vartheta ,\mathrm{Mb}\right)$ be the encryption key of the secret sharing algorithm, and let I(h,w)∈[0,255] be the original pixel values. To ensure that the split shadow images still take the form of images, with a maximum single-channel pixel value of 255, the elements of $M_{b}$ must satisfy the following:

(41) $$\begin{array}{c} \left\{128<Mb^{\left(1\right)}<Mb^{\left(2\right)}<\cdots <Mb^{\left(n\right)}\leq 251<Mp\right\}\\ \gcd\left(Mb^{\left(i\right)},Mb^{\left(j\right)}\right)=1,i\neq j\\ \gcd\left(Mb^{\left(j\right)},Mp\right)=1,i=1,2,\ldots ,n\\ \gcd\left(Mb^{\left(i\right)},\vartheta \right)=1,i=1,2,\ldots ,n \end{array}$$

Assuming the image owner sets a (*k*,*n*) threshold during the key generation stage, xϑ must satisfy the following relationship to achieve the threshold property:

(42) $$\prod_{i=1}^{k-1}Mb^{\left(i\right)}<x\vartheta <\prod_{i=1}^{k}Mb^{\left(i\right)}$$

However, the size of the pixel values *x* changes after fine encryption. Therefore, different random ϑ must be set to achieve a dynamically compatible threshold scheme when setting different values of *k*:

(43) $$\prod_{i=1}^{k-1}Mb^{\left(i\right)}/x<\vartheta <\prod_{i=1}^{k}Mb^{\left(i\right)}/x$$

This allows for (k,n) threshold secret sharing. Thus, definition 3 is proved. □

## 4. Experiments and Numerical Results

The six test images, including “Lena”, “Baboon”, “Barbara”, “Goldhill”, and “Peppers”, are from the dataset used for the experiments. All test images are grayscale images with a size of 512 × 512. The experimental environment was as follows: host configuration CPU AMD 5800X, memory 32 GB, operating system Windows 11, programming language python 3.9, and MATLAB 2022b.

### 4.1. Performance of Image Encryption

According to the guideline [28], we conduct multiple experiments to ensure the security of image encryption by testing image randomness, similarity metrics, graphic correlation, and noise robustness.

**Figure 7:**
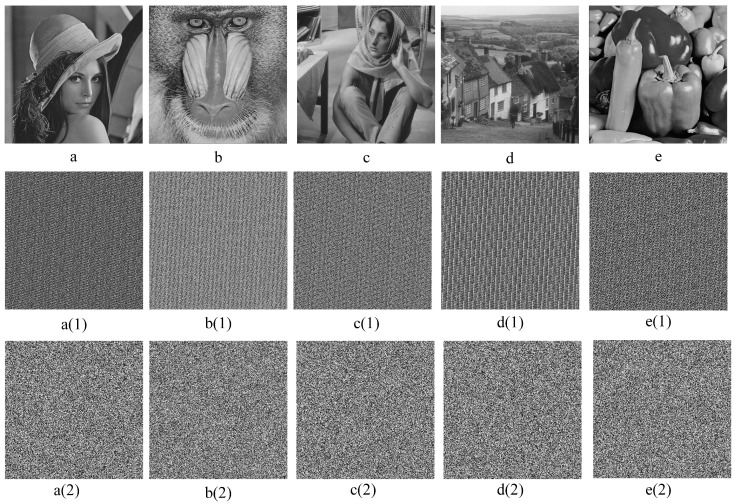
The visual effection of image encryption. The first row (**a**–**b**) is the original carrier image, the second (**a(1)**–**e(1)**) is the image after coarse-grained encryption, and the third (**a(2)**–**e(2)**) is the image after fine-grained encryption.

**Image randomness**. The intruder shares two secret images at the same time. To the naked eye, the shadow image must look like noise. In addition, the shadow images can be evaluated in a histogram; the more uniform the histogram distribution, the more secure the proposed scheme is. As shown in Figure 7, the first line is the encryption effect of Lena, Baboon, Barbara, Goldhill, and Peppers, and the second line is the encryption result after coarse-grained encryption, which exhibits a strong regularity among pixels, but it is visually impossible to tell what the original image is. The third line is the encryption result after fine-grained encryption. From the line, we can see that after the fine-grained encryption, the statistical features of the gray pixels of the carrier image can be effectively concealed.

In addition, we propose two evaluation metrics for the similarity metric, which can be measured by the peak signal-to-noise ratio (PSNR) shown in Equation (44) and the structural similarity (SSIM) shown in Equations (45) and (46). PSNR evaluates the similarity of images and MSE denotes mean square error:

(44) $$\left\{\begin{array}{c} PSNR=10\times \log_{10}\left(\frac{255^{2}}{MSE}\right)dB\\ MSE=\frac{1}{W\times H}\sum_{i=1}^{W}\sum_{j=1}^{H}{\left[S^{\prime }\left(i,j\right)-S\left(i,j\right)\right]}^{2} \end{array}\right.$$

(45) $$\mathrm{SSIM}\left(S,S^{\prime }\right)=\frac{\left(2\mu_{S}\mu_{S^{\prime }}+c_{1}\right)\left(2\sigma_{SS^{\prime }}+c_{2}\right)}{\left(\mu_{S}^{2}+\mu_{S^{\prime }}^{2}+c_{1}\right)\left(\sigma_{S}^{2}+\sigma_{S^{\prime }}^{2}+c_{2}\right)}$$

(46) $$\left\{\begin{array}{c} c_{1}={\left(0.01\times L\right)}^{2},c_{2}={\left(0.03\times L\right)}^{2},\\ \left\{x,y|S,S^{\prime }\;\mathrm{and}\;x\neq y\right\},\left\{L|0\leq L\leq 255\right\},\\ \mu_{x}=\frac{1}{W\times H}\sum_{i=1}^{W}\sum_{j=1}^{H}x\left(i,j\right),\sigma_{x}=\sqrt{\sigma_{x}^{2}}\\ \sigma_{x}^{2}=\frac{1}{W\times H-1}\sum_{i=1}^{W}\sum_{j=1}^{H}{\left(x\left(i,j\right)-\mu_{x}\right)}^{2},\\ \sigma_{xy}=\frac{1}{W\times H-1}\sum_{i=1}^{W}\sum_{j=1}^{H}\left(x\left(i,j\right)-\mu_{x}\right)\left(y\left(i,j\right)-\mu_{y}\right) \end{array}\right.$$

**Figure 8:**
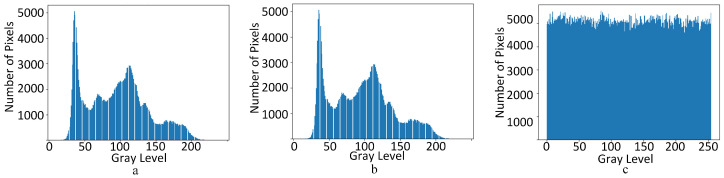
The gray histogram of Lena. From left to right are the original carrier image (**a**), the coarse-grained encrypted image (**b**), and the fine-grained encrypted image (**c**).

Furthermore, we use the one-dimensional grayscale entropy of an image to the amount of average information in an image. The one-dimensional entropy indicates the amount of information contained in the aggregated features of grayscale distribution in the image so that $p_{i}$ denotes the proportion of pixels with a grayscale value *i* in the image. The one-dimensional grayscale entropy of a grayscale image is defined as, for an image, the closer the information entropy is to 8, the more confusing the image is. As can be seen from Table 1, the information entropy of the images after fine-grained encryption can reach 7.999 information entropy, indicating that the proposed encryption effect can meet the security requirements. As shown in Figure 8, the histogram also shows that the statistical properties of the original pixels are effectively masked after encryption, which has statistically solid properties before fine-grained encryption:

(47) $$H=\sum_{i=0}^{255}p_{i}\log p_{i}$$

**Graphic Correlation.** Pixel values in plain images are highly similar to their neighbors, whether located horizontally, vertically, or diagonally. This strong correlation poses a risk for statistical attacks, necessitating efficient cryptosystems to create non-correlated encrypted images. Graphic correlation is a visual inspection of an image’s pixel correlation, with the horizontal axis indicating pixel intensity and the vertical axis showing neighbor pixel values.

**Figure 9:**
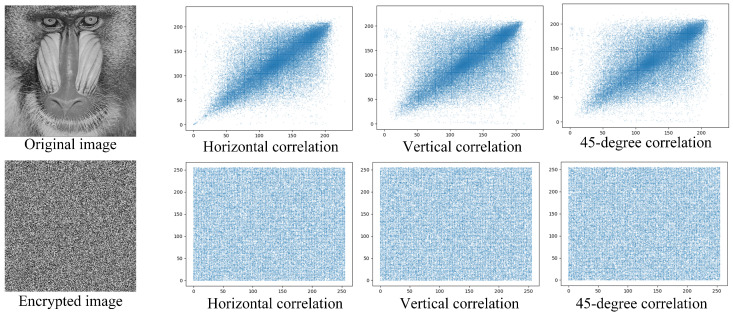
Results of the graphic correlation test in the horizontal, vertical, and 45-degree directions.

As shown in Figure 9, we conducted thorough experiments to validate the correlation between pixels. We performed a block-by-block analysis of the graphic correlation of both the original image and the image encrypted with our fine-grained encryption system. The results revealed a significant similarity between the pixel values of the original image and its neighboring pixels, creating a severe vulnerability to statistical attacks. Additionally, the correlation pattern over the 45-degree line in the original image was observed to be strong. The closer the pattern was to this line, the higher the pixel correlation. Nevertheless, we observed that our fine-grained encryption system generated an encrypted image. A visual inspection of its graphic correlation revealed strong non-correlation in the horizontal, vertical, and 45-degree directions. This is due to most neighboring pixels possessing different intensity values.

**Figure 10:**
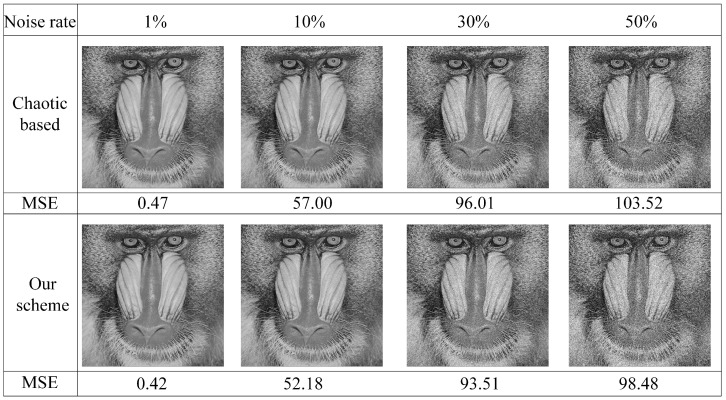
Noise robustness: The visual effects under different noise rates (1%, 10%, 30%, 50%) are shown through the MES results, which demonstrate the stronger ability of the proposed algorithm to resist noise attacks.

**Noise Robustness**. The transmission channel may cause encrypted images to suffer from noise attacks or interference, making it difficult to recover the plain image. Therefore, evaluating noise robustness is necessary for chaos-based image cryptosystems. Our selected algorithms employ substitution–permutation networks, stream encryption, and one-per-one pixel encryption, providing certain advantages against noise. With these principles and bulk image data, the original plain image can still be reconstructed with high visual quality even during the encryption process. To further enhance the noise robustness of our method, we have adopted an error correction algorithm. In this algorithm, if the first pixel value of a 2 × 2 image block is greater than the second pixel value, the second pixel value is assigned to the first pixel. Similarly, if the third pixel value is greater than the fourth pixel value, the fourth pixel value is assigned to the third pixel. We test the visual effects of our proposed algorithm under different levels of noise (1%, 10%, 30%, 50%) to demonstrate its ability to resist noise attacks, as shown in Figure 10. The test results indicate that our proposed algorithm with error correction capability has a stronger resistance against noise attacks than the recovery based on pure chaotic systems, as indicated by the MES results.

**Figure 11:**
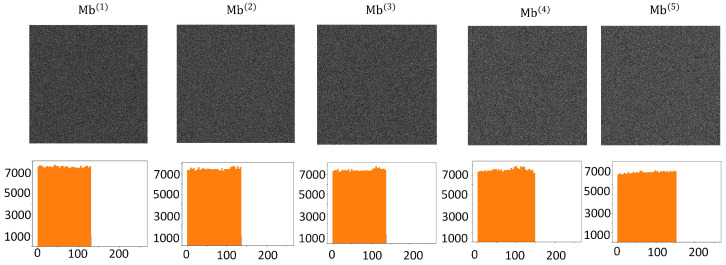
Comparison of statistical histograms of shadow images under different modules.

**The randomness of shadow images**. Assuming that an attacker can share two shadow images simultaneously, both must be indistinguishable noise classes. In addition, shadow images can be evaluated in histograms; the more uniform the distribution of the histograms, the safer the proposed scheme. As can be seen from Figure 11, the histogram distribution is approximately the same under different module bases, and no valid information can be distinguished from the shadow image.

**Figure 12:**
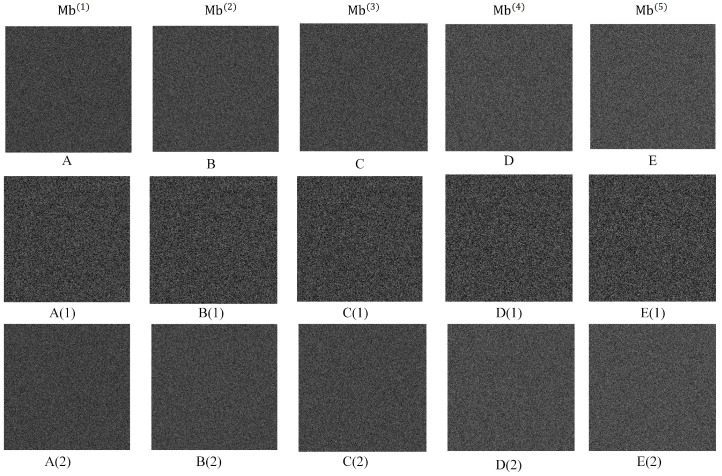
Visual display of the shadow images, guide images, and embedded images. The first row (**A**–**E**) is the shadow image, the second row (**A(1)**–**E(1)**) is the embedded guide image, and the third row (**A(2)**–**E(2)**) is the shadow image after embedding the secret message statistical histograms of shadow images under different modulus bases.

Figure 12 shows the shadow image under different module bases and the data obtained by embedding the secret key. It can be seen that the shadow image is sufficiently able to show the correlation between pixels. For the convenience of the display, the pixel box in the upper right corner corresponds to the pixel value of the first 2 × 2 sub-block in the upper leftmost corner. It can be seen that each data hider recovers a different guide image, but all generate the same different histogram.

### 4.2. Comparison with the State of the Art

To better measure our proposed method, we introduce an embedding rate formula. In order to show the embedding strategy more clearly, the embedding rate used to evaluate the embedding capacity of the proposed scheme and other related schemes is calculated as follows:

(48) $$ER\left(bpp\right)=\frac{\;\mathrm{Total}\;\mathrm{number}\;\mathrm{of}\;\mathrm{embedded}\;\mathrm{bits}\;\mathrm{of}\;\mathrm{the}\;\mathrm{image}\;}{H\times W}$$

It can be seen that the embedding capacity is related to the embedding step size and embedding size. The larger the step size, the larger the embedding capacity, and the more pronounced the influence on the visual effect.

**Figure 13:**
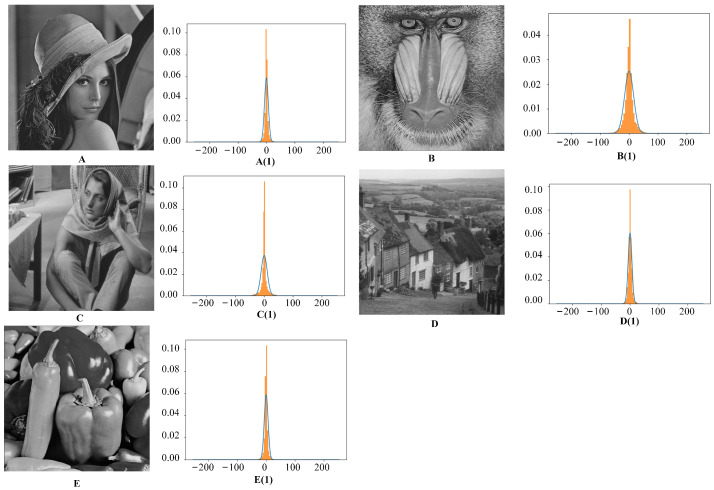
Comparison of pixel difference histograms of different images. Images (**A**–**E**) represent the original images, while image (**A(1)**–**E(1)**) correspond to the difference histograms of the encrypted images.

The statistical histograms of the ciphertext prediction errors for five images are shown in Figure 13. Since the plaintext prediction error and ciphertext have the same statistical error, we can use the histogram transfer formula Equations (17) and (18) to hide the data in the ciphertext images. In addition, the prediction error histograms of the ciphertext images of “Lena”, “Goldhill”, and “Peppers” are mainly around the value of 0, while the prediction difference histograms of Baboon and Barbara are far from the value of 0 of the three images mentioned above. It can also be seen from the table that Baboon and Barbara are different from “Lena”, “Goldhill”, and “Peppers" in terms of having lower embedding rates.

Table 2 shows the theoretical embedding rate/actual embedding rate. We conducted tests on the actual embedding rate by employing Lena plots and set the three-out-of-four threshold. It is known from the algorithm proposed in this paper that, for each block, the pixel corresponding to the maximum value and the pixel corresponding to the minimum value of the four-pixel blocks can perform embedding, i.e., half of the pixels can perform embedding after deciding whether the value in the pixel can be embedded in the data according to different thresholds. In addition, the size of the embedding amount should be less than the threshold value, and this paper uses the embedding amount of bits 1, 2, 3 corresponding to the threshold bits 1, 2, 3, 4, respectively. The table shows that the embedding rate is obtained at 1.038, 2.316, and 5.226, depending on how the embedding amount and threshold bits are selected. It can be seen that the proposed method in this paper is linear in the growth of the embedding amount. This growth is in line with the increase in the corresponding embedding amount and the number of threshold bits.

We compare the embedding rate of the proposed method with several state-of-the-art methods. As shown in Table 3, the embedding rate of the proposed method is significantly higher than those of the prior art methods. Furthermore, the embedding rate of the method varies with the different histograms of the image. The closer the value in the difference histogram is to zero, the higher the embedding rate is. Images with smooth textures can obtain higher embedding rates, while images with complex textures have lower embedding rates, such as the image Baboon.

This paper and other methods exploit the correlation of natural images, such as MSB prediction [10,11], and differential extension [23], all of which have embedding rates that vary with the redundancy of the images. In addition, the method proposed in this paper does not require data compression. However, it performs data embedding by recovering histograms that retain pixel difference information through the adaptive generation of bootstrap maps that automatically generate embedding secret keys, from which high embedding rates are obtained.

In the scheme comparison, as shown in Table 4, all the compared algorithms use the Vacating room approach to achieve an embedding efficiency higher than 0.5 bpp. Among them, [10,11,23] use the Vacating room approach before encryption to increase the embedding capacity. In addition, the proposed method in this paper uses a combination of stream encryption and secret sharing based on chaos, which is more secure than other state-of-the-art algorithms, single stream encryption [10,11], and single secret sharing [23]. In addition, this paper innovatively proposed a way of generating guidance graphs by shadow images, which reduces the scheme’s complexity and the variety of information transfer.

## 5. Conclusions

This study addresses several key issues, including reversible information hiding in encrypted domains in various data-embedding scenarios, improved encryption effectiveness, and enhanced information-embedding capability. We propose a lossless RDH-EI method based on PVO and secret sharing for multiple data hiders, which addressed the problem of secure and efficient data embedding in encrypted images. The method ensures lossless embedding and extraction, and the original image’s digital assets can be fully recovered. The proposed PVO-based secret sharing scheme achieves a high embedding rate and is significantly better than existing encryption techniques in terms of efficiency and effect. The combined stream encryption with the secret sharing approach significantly improves the security of the method, ensuring that the data hiders can embed their data securely without any fear of data leakage. Moreover, the proposed scheme does not require any extra guide parameters, which enhances its usability.

## Figures and Tables

**Table 1:** PSNR, entropy, and SSIM for the encrypted image.

Test Images	Coarse-Grained Encrypted Image			Coarse-Grained Encrypted Image			Restored Image		
	PSNR (dB)	Entropy	SSIM	PSNR (dB)	Entropy	SSIM	PSNR (dB)	Entropy	SSIM
Lena	12.272	7.234	0.030	8.913	7.999	0.011	*∞*	7.234	0
Baboon	12.614	7.358	0.032	9.526	7.999	0.011	*∞*	7.358	0
Barbara	11.666	7.466	0.029	9.104	7.999	0.012	*∞*	7.466	0
Goldhill	11.28	7.478	0.0327	9.040	7.999	0.011	*∞*	7.4777	0
Peppers	9.942	7.571	0.0182	8.430	7.999	0.012	*∞*	7.571	0

**Table 2:** Embedding rate of proposed scheme.

Embedding Amount	Theoretical Embedding Rate				Actual Embedding Rate			
	**1 bit**	**2 bit**	**3 bit**	**4 bit**	**1 bit**	**2 bit**	**3 bit**	**4 bit**
1 bit	0.5	1.5	1.5	1.5	0.346	1.038	1.305	1.419
2 bit	-	3	3	3	-	2.316	2.613	2.838
3 bit	-	-	6	6	-	-	5.226	5.679

**Table 3:** Embedding rate among the proposed scheme and state-of-the-art schemes.

Image Name	[10]	[11]	[23]	[29]	[30]	Ours
Lena	0.973	1.575	0.5	3.5	5.3	**5.676**
Baboon	0.872	0.728	0.5	3.5	**5.3**	4.776
Barbara	0.96	1.265	0.5	3.5	5.3	**5.367**
Goldhill	0.963	1.542	0.5	3.5	5.3	**5.583**
Peppers	0.976	1.545	0.5	3.5	5.3	**5.703**
Average	0.959	1.331	0.5	3.5	5.3	**5.421**

**Table 4:** Feature comparison among the proposed scheme and state-of-the-art schemes.

Scheme	Separable	Vacating Room	Encryption Strategy	Multi-Data Hider	Availability of Guidance Information
[10]	Yes	Before	Stream cipher	No	Yes
[11]	Yes	Before	Stream cipher	No	Yes
[23]	No	Before	Secret sharing	No	Yes
Ours	**Yes**	**Before**	**Stream cipher+ Secret sharing**	**Yes**	**No**

## Data Availability

Not applicable.
